# Amiloride Enhances Antigen Specific CTL by Faciliting HBV DNA Vaccine Entry into Cells

**DOI:** 10.1371/journal.pone.0033015

**Published:** 2012-03-16

**Authors:** Shuang Geng, Yiwei Zhong, Shuang Wang, Hu Liu, Qiang Zou, Xiaoping Xie, Chaofan Li, Qingling Yu, Zhonghuai He, Bin Wang

**Affiliations:** 1 State Key Laboratory for Agro-Biotechnology, China Agricultural University, Beijing, China; 2 Key laboratory of Medical Molecular Virology of MOH and MOE, Shanghai Medical College, Fudan University, Shanghai, China; 3 Beijing Advaccine Biotechnology, Beijing, China; University Hospital Zurich, Switzerland

## Abstract

The induction of relatively weak immunity by DNA vaccines in humans can be largely attributed to the low efficiency of transduction of somatic cells. Although formulation with liposomes has been shown to enhance DNA transduction of cultured cells, little, if any, effect is observed on the transduction of somatic tissues and cells. To improve the rate of transduction, DNA vaccine delivery by gene gun and the recently developed electroporation techniques have been employed. We report here that to circumvent requirement for such equipment, amiloride, a drug that is prescribed for hypertension treatment, can accelerate plasmid entry into antigen presenting cells (APCs) both in vitro and in vivo. The combination induced APCs more dramatically in both maturation and cytokine secretion. Amiloride enhanced development of full CD8 cytolytic function including induction of high levels of antigen specific CTL and expression of IFN-γ+perforin+granzymeB+ in CD8+ T cells. Thus, amiloride is a facilitator for DNA transduction into host cells which in turn enhances the efficiency of the immune responses.

## Introduction

DNA vaccination first proved to be effective in the 1990s when it was used to treat viral infection [1], [2]. It was shown to favor cellular immune responses in contrast to recombinant subunit vaccines that favor humoral responses [3], [4]. However, unsuccessful clinical trials indicated that DNA vaccines suffered from low efficiency in transducing host cells via syringe-based delivery [5]. A great improvement in transduction was obtained by using DNA plasmid that was coated on gold pellets and bombarded into somatic cells by using gene gun technology [6], [7], [8], [9]. Recently, even higher transduction efficiency has been achieved by the use of in vivo electroporation devices [10], [11], [12], [13]. Both approaches require special equipment and may cause some discomfort in vaccinees [14]. Although there have been many other approaches to enhance efficiency, including the use of adjuvants, cytokines, nanoparticles etc., few approaches have focused on enhancing transduction of somatic cells. The mystery of why liposome delivery of DNA into cultured cells is very efficient, but the same delivery into cells in vivo is inefficient has not been completely solved. Although some chemical compounds, such as Bupivacain [15], have been shown to enhance DNA entry into muscle when given in pretreatment, directly enhancing DNA uptake into somatic cells remains a challenge.

Amiloride, an inhibitor of the Na/K pump of cellular membranes [16], has been routinely used as an inhibitor of macropinocytosis [17]. Due to this effect, it has been clinically prescribed to treat hypertension[18]. No report has been made of an effect on DNA plasmid transduction of cells or tissues or of subsequent effects on the immune responses.

Here we report that amiloride effectively accelerates DNA entry into cells *in vitro* and *in vivo*, with subsequent adjuvancity on both innate and adaptive responses, particularly on antigen specific CD8^+^ CTL responses. Therefore, amiloride might be further developed as a new facilitator for DNA vaccines.

## Results

### Amiloride accelerates DNA entry into APC

**Figure 1:**
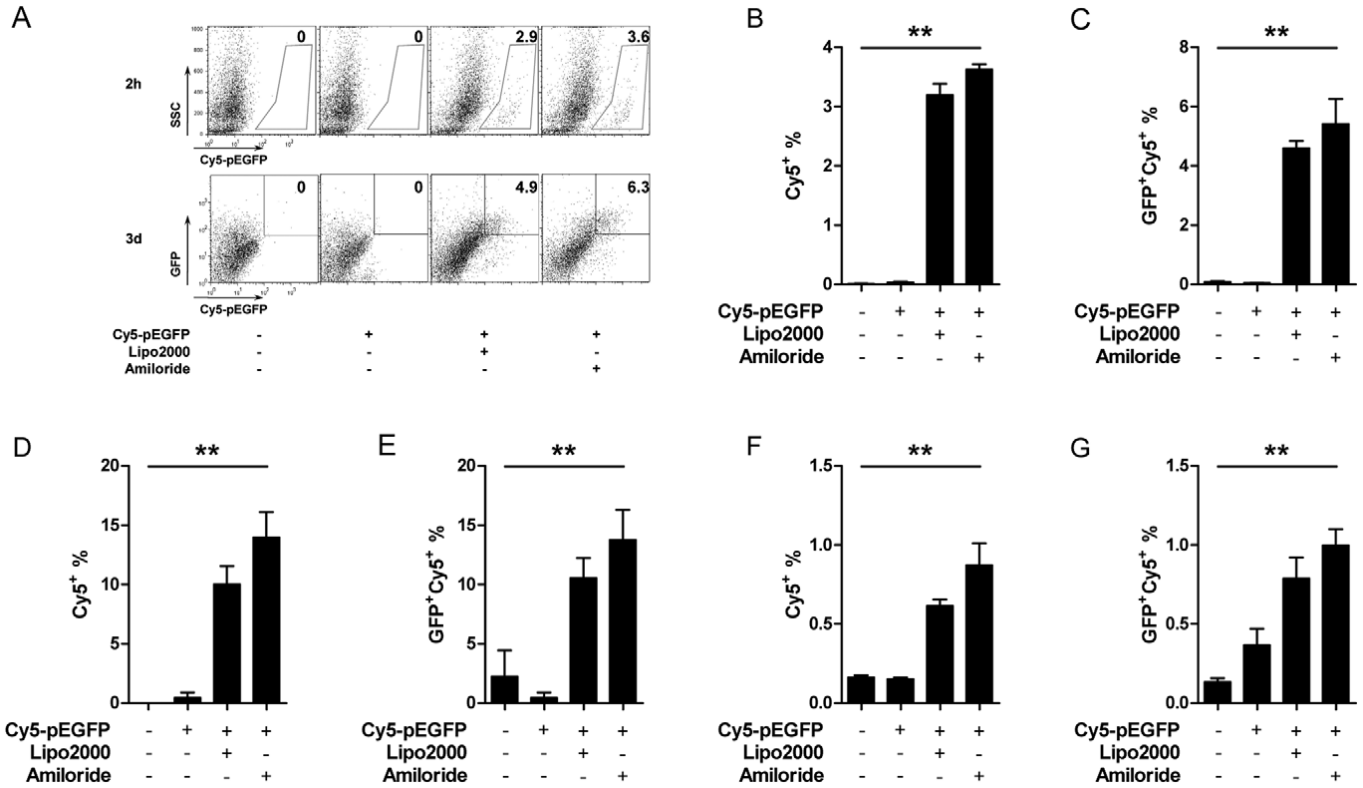
Amiloride facilitates plasmid entry *in vitro*. Cy5-pEGFP entry into cell lines with or without 1 mM amiloride was analyzed after 2 h and is shown as the percentage of cells that were Cy5^+^. Expression of GFP was analyzed at day 3 and is shown as percentage of EGFP^+^Cy5^+^, on RAW264.7 (A, B, C), JAWSII (D, E), and DC2.4 (F, G). Data represent one of three independent experiments.

We initially observed that amiloride enhanced DNA entry into the JAWSII DC cell line when we performed endocytosis inhibition assays (Data not shown). This phenomenon was again observed with both a macrophage cell line (RAW264.7) and dendritic cell lines (JAWSII and DC2.4). When the cells were pre-treated with 1 mM amiloride for 1 h, we observed that uptake of Cy5-labeled pEGFP plasmids was significantly enhanced within 2 hrs and the cells expressed a significantly higher level of GFP after 3d culture compared with the un-treated cells. This high level of expression was similar to that seen when DNA was delivered with liposomes (Fig. 1).

**Figure 2:**
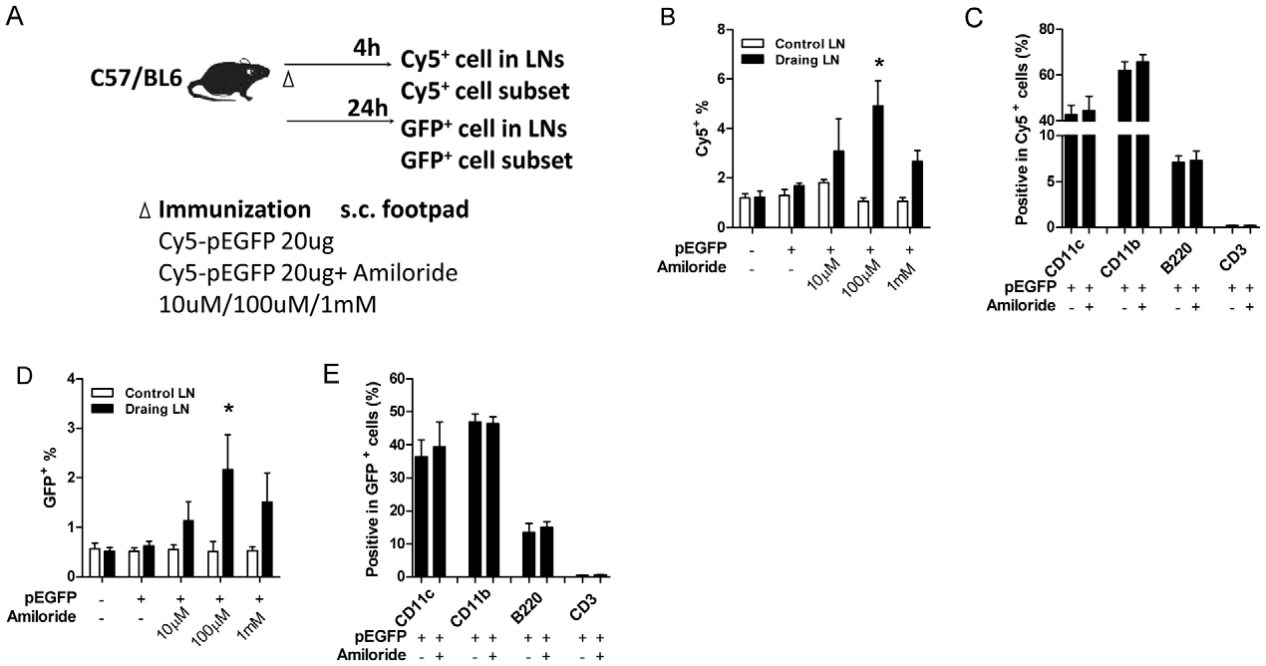
Amiloride accelerates plasmid entry *in vivo*. Naïve C57 mice (n = 3) were immunized with Cy5-pEGFP *s.c.* in hind footpad with (right side) or without (left side) amiloride. Lymph node cells were collected after 4 h (Cy5^+^ cells) and after 24 h (GFP^+^ cells) and examined for proportion (B, D) and subtype (C, E) of stained cells from both draining and control lymph nodes. * in B and D, statistical significance between control and draining lymph nodes in all treated groups.

Although amiloride could facilitate DNA plasmid entry into cultured cells, we considered that it might not work with somatic cells. To test this *in vivo,* Cy5-labeled pEGFP plasmid, with or without amiloride, was injected into the hind footpads of C57Bl/6 mice. After 4 hrs, draining lymph nodes were collected and Cy5^+^ cells were analyzed by FACS (Fig. 2A). The inguinal lymph nodes from the un-injected side were also collected as negative controls. The percentage of Cy5-plasmid^+^ cells in LN was increased with 10 µM amiloride, peaked at 100 µM, but decreased at 1 mM (Fig. 2B). We next analyzed whether transfected cell subset was affected by amiloride. Data showed that as amiloride accelerated cell transfection, cell subset remain unaltered: the majority of Cy5^+^ cells were CD11c^+^ and CD11b^+^, suggesting dendritic cells and macrophages, and ∼10% was positive for B220, a B cell marker; but few were T cells since only a background signal was obtained after CD3^+^ staining (Fig. 2C). The facilitated cell entry also resulted in higher levels of transduced gene expression as demonstrated by the higher GFP intensities after 24 h, and similar transfection of cell subsets. (Fig. 2D and 2E).

DNA entry into cells was previously reported to be through endocytosis [19], [20], [21]. To examine which endocytic pathway mediated this entry, we used specific endocytosis inhibitors to see their effects. Adding either MβCD, an inhibitor of lipid-raft dependent endocytosis, or fillipin, an inhibitor of caveolae-dependent endocytosis, reduced the amiloride-mediated DNA entry and gene expression. The amiloride-mediated DNA entry could be completely abolished by MβCD plus fillipin in RAW264.7 cells (Fig. S1A, B). Similar inhibition was also observed in both JAWSII and DC2.4 cell lines (Fig. S1C, D, E, F). These results suggest that amiloride mediated DNA entry is through both lipid-raft- and caveolae- dependent endocytosis *in vivo*.

### Amiloride enhances innate immunity

**Figure 3:**
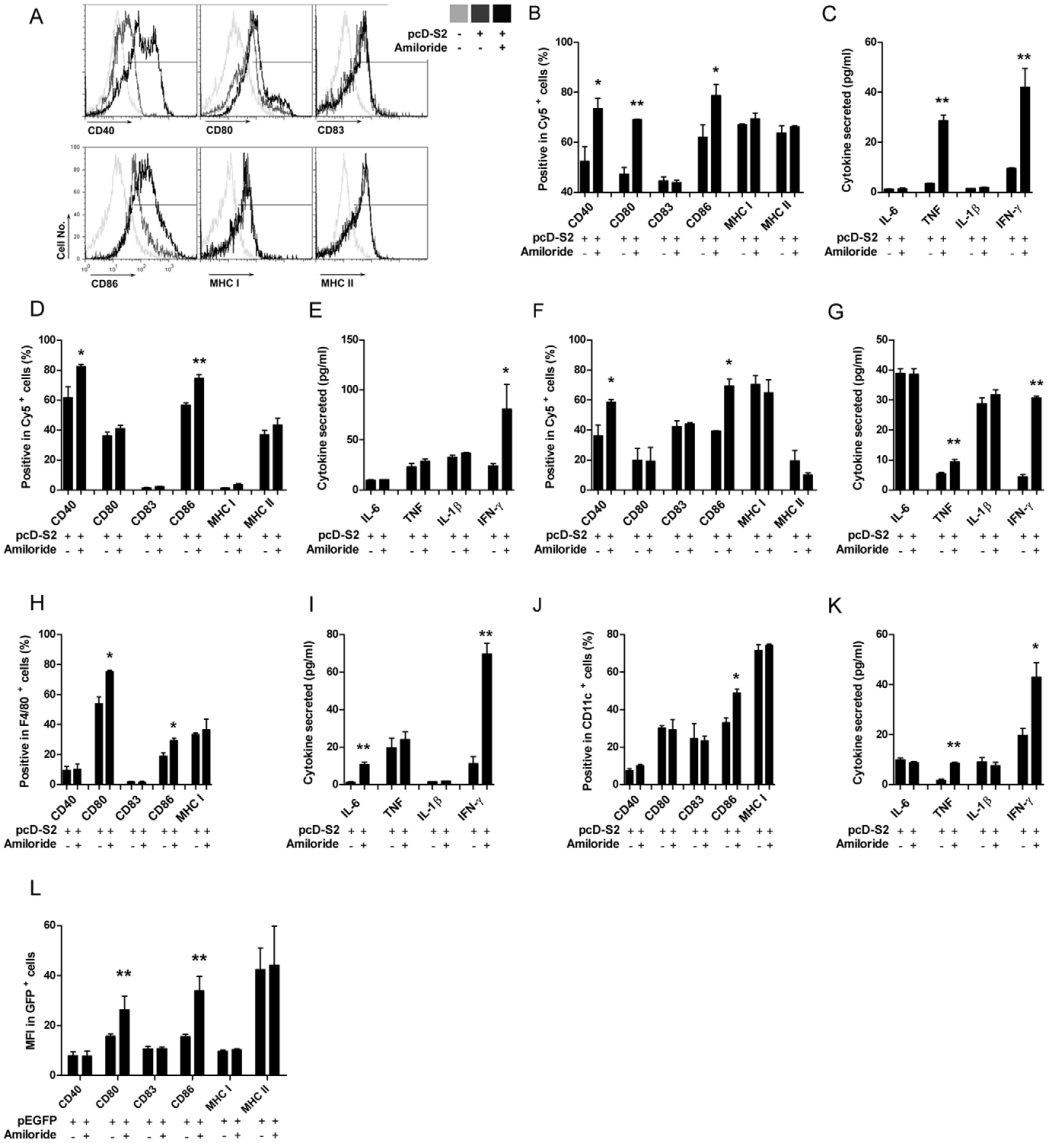
Amiloride enhances APC maturation and innate cytokine secretion. pcD-S2 at 10 µg/ml with or without 1 mM of amiloride was added in cell culture for stimulation. Surface maturation markers CD40, CD80, CD83, CD86, MHC-I, MHC-II and secreted innate cytokines IL-6, TNF-α, IL-1β, IFN-γ in supernatant, were tested at day 3 on RAW264.7 (A, B, C), JAWSII (D, E), DC2.4 (F, G), peritoneal macrophage (H, I) and spleno-DC (J, K). pEGFP was injected into footpad with or without amiloride, 24 h later, surface maturation markers were analyzed on GFP+ cells from draining LN (L). Shown is one of three independent experiments with similar results, analyzed based on Cy5+ (A, D, F), F4/80+ (H), CD11c+ (J) or GFP+ (L)cells. For peritoneal macrophage and spleno-DC, n = 3. * and ** indicate significant difference between +/− amiloride.

Because amiloride facilitated the entry of DNA into, and subsequent higher gene expression in antigen presenting cells (APCs), we asked if this could positively affect innate immune responses. To test this notion, after verification of amiloride alone has no effect on innate immune response (Fig. S2), we used a HBV DNA vaccine, pcD-52, encoding HBsAg and conjugated with Cy5 as a model. Higher levels of expression of CD40, CD80 and CD86 were obtained on cultured RAW264.7 cells (Fig. 3A, B and Fig. S3A), suggesting that amiloride treatment could increase the level of maturation for this macrophage cell. Consistent with enhanced maturation, we also observed that higher levels of expression of TNF and IFN-γ were induced with amiloride treatment than without treatment (Fig. 3C). Similar enhanced maturation status was reached in both dendritic cell lines, DC2.4 and JAWSII, although there were some differences in expression levels for the pro-inflammatory cytokines (Fig. 3D, E, F, G and Fig. S3B, C).

Since cell lines can have skewed cytokine profiles caused by prolonged culture, we next tested the treatments with freshly isolated APCs, either peritoneal macrophages or splenic dendritic cells. Both cell types showed higher expression of maturation markers and more pro-inflammatory cytokine secretion when the cells were treated with pcD-S2 plus amiloride than with pcD-S2 alone (Fig. 3H, I, J, K and Fig. S3D, E).

To confirm amiloride's up-regulation of innate immunity *in vivo*, we injected pEGFP into right hinder footpad, with or without amiloride, and analyzed maturation markers of transduced (GFP^+^) cells. Again, data showed CD80 and CD86 were up-regulated by amiloride treatment (Fig. 3L).

### Amiloride enhances immune responses including strong CMI for pcD-S2 DNA vaccine

**Figure 4:**
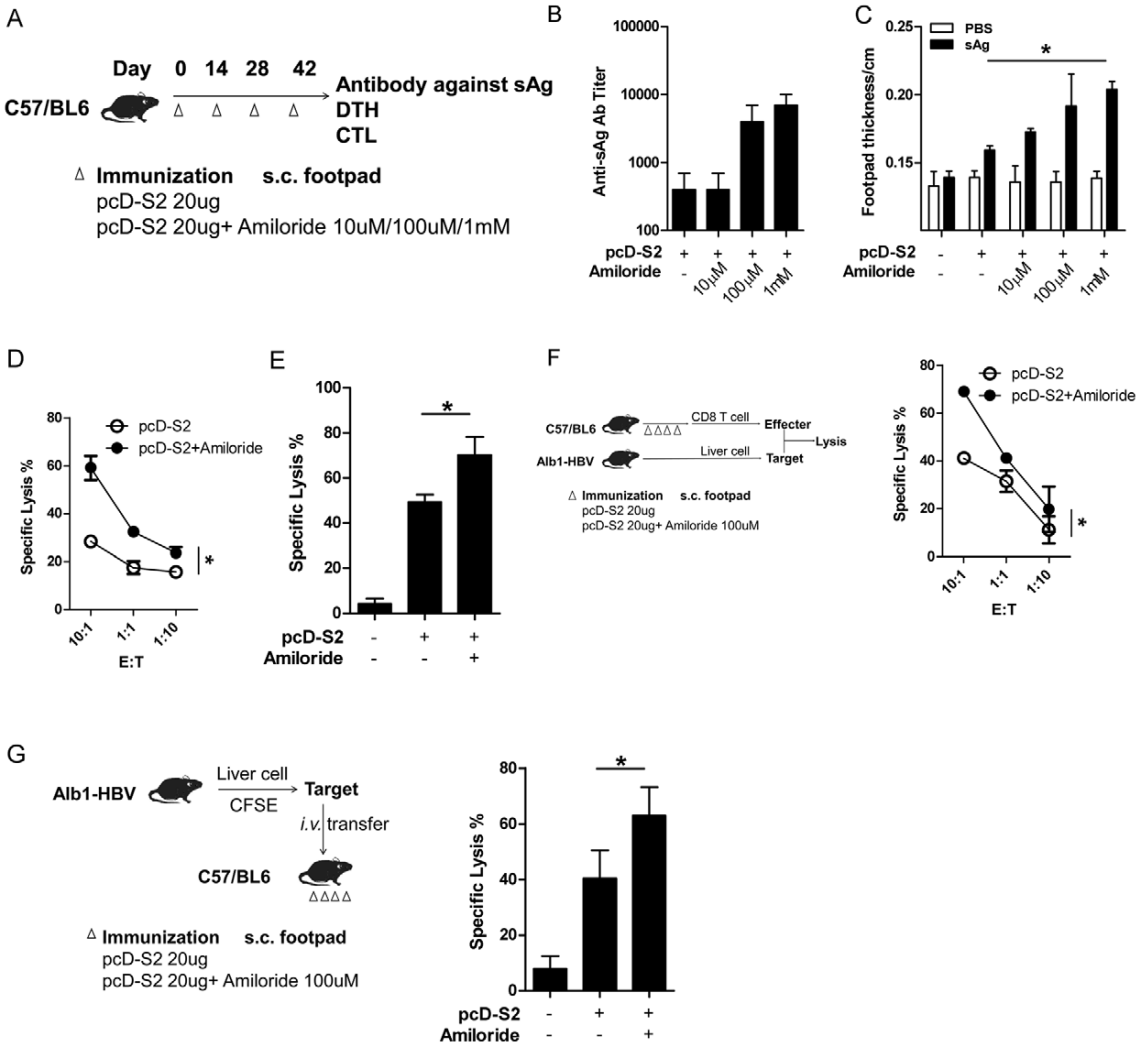
Amiloride enhances adaptive immunity against HBV S2. Naïve C57 mice were immunized s.c. with pcD-S2 at various concentrations with or without amiloride in the hind footpad four times using the immunization scheme as shown (A). Seven days after the last immunization, animals (n = 3) were used to test anti-S2 IgG antibody titer (B) and delayed hypersensitivity (DTH) response after re-stimulation with 1 µg sAg *s.c.* in a hind footpad for 24 h (C). PBS was added as negative control. *, statistical significance among all groups. Another 3 animals were used to test HBV S208–215 specific lysis *in vitro* (D) and *in vivo* (E). Hepatocytes from HBV Alb1 trangenic mice were used as targets mixed with effectors from the immunized mice, or transferred into the immunized mice before the analysis of specific lysis *in vitro* (F) and *in vivo* (G). *, statistical significance between +/− amiloride.

Enhancement of innate responses may affect adaptive responses. To test this, plasmid DNA, pcD-S2, with or without amiloride, was injected into footpads of C57B/6 mice (Fig. 4A). Antibody against HBsAg was increased in the amiloride+ group compared to groups given pcD-S2 alone and the effect was dose-dependent (Fig. 4B). The DTH reaction against HBsAg was also increased more in the pcD-S2 plus amiloride groups than those given pcD-S2 alone (Fig. 4C). Both experiments showed that 1 mM of amiloride was the most effective dose for *in vivo* treatment.

DTH reflects cell mediated immunity (CMI), of which an important component is the CD8+ cytolytic T lymphocyte (CTL). To explore if amiloride could also influence CTL, CD8+ T cells from immunized mice were purified as effector cells, and naïve C57 splenocytes were treated with HBsAg peptide S208–215 and subsequently labeled with CFSE for use as target cells. The cells were mixed at different effector/target ratios. After 3 days culture at E:T ratio of 10∶1, 60 percent of target cells were lysed in the amiloride plus pcD-S2 group, significantly higher than ∼30% lysis in the pcD-S2 alone group (Fig. 4D). Furthermore, *in vivo* CTL assay was performed by using peptide treated CFSE labeled target cells that were transferred into immunized syngeneic mice via i.v. injection. A stronger cytolytic activity was achieved in the amiloride plus pcD-S2 group compared with the untreated counterparts (Fig. 4E).

This antigen specific killing was further demonstrated in the use of liver cells from Alb1-HBV mice, liver-specific HBsAg transgenic mice, as target cells in the in vitro test and in the in vivo test (Fig. 4F, G). Consistent with previous results, a higher level of CTL was achieved in the amiloride plus pcD-S2 group compared to the controls.

### Amiloride increases triple positive CD8 T cells

**Figure 5:**
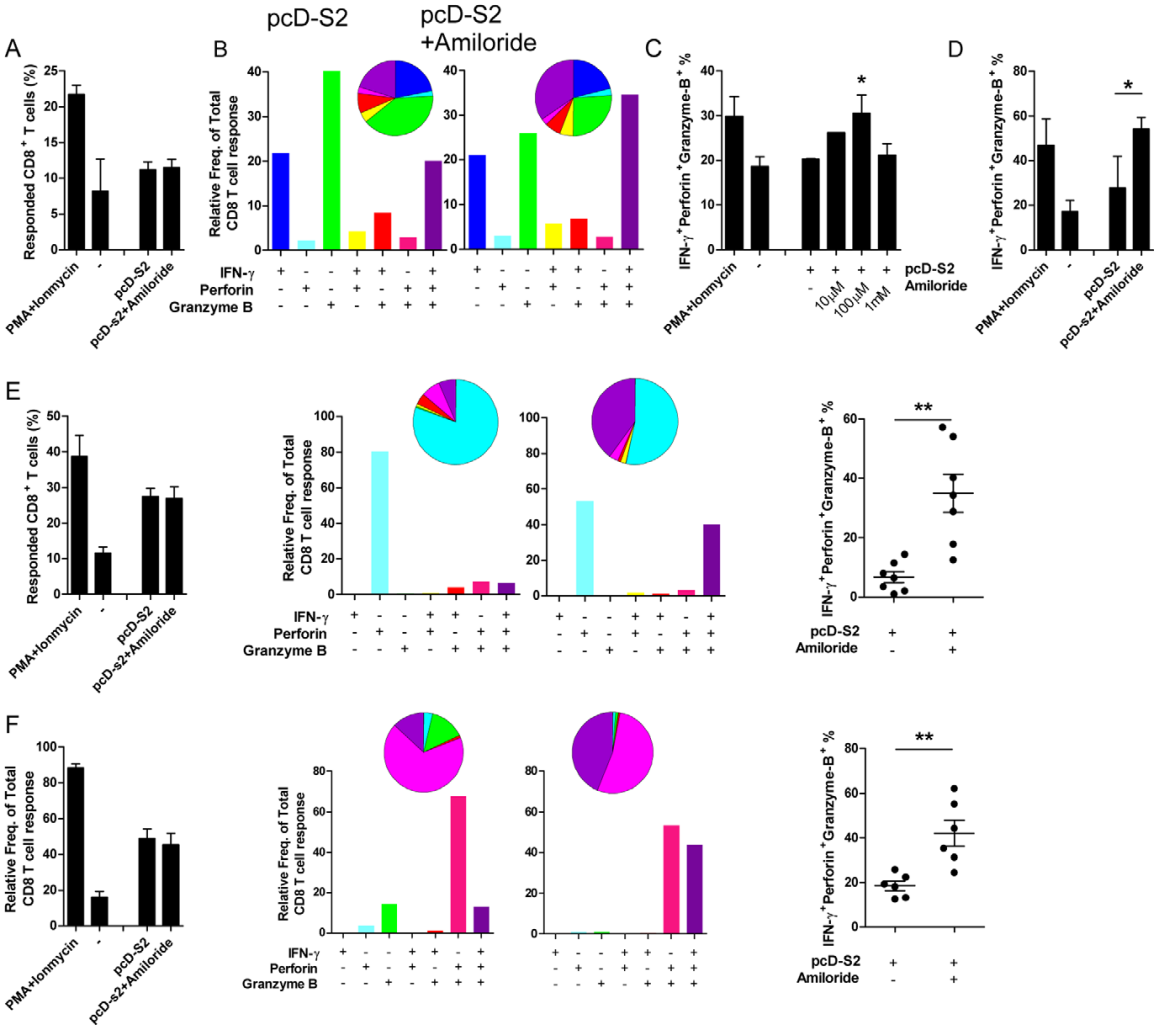
Amiloride increases the frequency of triple positive CD8 T cells. Splenocytes from mice immunized with pcD-S2 with or without amiloride (n = 3) were re-stimulated in vitro with 10 µg/ml S208–215 for 12 h (A-C), or 10 µg/ml HBsAg for 24 h (D), then cytokine secretion was blocked by monensin for 6 h. PMA or Ionomycin stimulating splenocyte of pcD-S2 immunized mice was added as positive controls. Cells stained with anti-CD3 and anti-CD8 were gated and then used for intracellular staining with single or multiple fluorescent-labeled antibodies. A shows the proportion of responsive cells in the total CD8^+^ T cells with or without amiloride treatment. These responsive cells were designated as either IFN-γ^+^, perforin^+^, or granzymeB^+^ cells. B shows the cytokine expression pattern in the responsive CD8 T cells with or without amiloride treatment. C shows the dose dependent effects of amiloride on the frequency of (IFN-γ^+^perforin^+^granzymeB^+^) triple positive cells. D shows the change in frequency of triple positive cells in response to HBsAg re-stimulation. The change in frequency of triple positive cells after co-culture with peritoneal macrophages followed by re-stimulation with S208–215 (E), or with spleno-DC (F) are also shown. Data represent three independent experiments with similar results.

IFN-γ, perforin and granzyme B are the essential components of CTL that contribute viral clearance [22], [23], [24], [25], [26]. The presence of these multiple factors within a single CD8 T cell, creating a multi-functional effector, has been documented as a more powerful anti-viral effector system [25], [27], [28], [29]. To test if amiloride has an adjuvant effect on multi-functional CTL, we performed a simultaneous FACS assay of multiple cell markers including IFN-γ, perforin and granzyme B to differentiate for cytolytic CD8+ T effectors (Fig. S4A). Compared with pcD-S2 immunization alone, immunization with amiloride plus pcD-S2 didn't increase the frequency of CD8+ T effectors responding to specific antigen (Fig. 5A). However it did increase the proportion of triple positive CD8+ T effectors within the responding CD8+ population (Fig. 5B, C and Fig. S4B). Furthermore, the increased proportion of triple positive cells could also be observed in an HBsAg-stimulated CD8 response (Fig. 5D), suggesting a more general boost by amiloride of CD8 T cell cytotoxity against HBV. Taken together, the results indicated that there would be stronger and more efficient killing of target cells.

To further demonstrate that the increase of triple positive CD8 T effectors was likely to be due to effects on APCs, peritoneal macrophages and spleen dendritic cells were collected and treated with pcD-S2 with or without amiloride, then co-cultured for 5 days with purified CD8 T cells from HBsAg immunized mice. During co-culture, epitope S208–215 was used to re-stimulate. Results showed that amiloride significantly increased the percentage of S208–215 specific triple positive CD8 T effectors in the co-culture system, both with macrophages (Fig. 5E and Fig. S4C) and DCs (Fig. 5F and Fig. S4D).

## Discussion

Here we report that amiloride, a previous prescribed drug for hypertension, could facilitate plasmid entry into antigen presenting cells (APCs), both in vitro and in vivo. Enhanced antigen expression in APC was accompanied by accelerated maturation and cytokine secretion. This led to enhanced adaptive immune responses as evidenced at the level of antigen specific CD8 T cytolytic function, which included induction of more highly effective antigen specific CTL, and the higher expression of IFN-γ^+^perforin^+^granzymeB^+^ in the antigen specific CD8^+^ T cells. Therefore, this study demonstrates that amiloride is a facilitator for DNA transduction into host cells, which in turn enhances the efficiency of immune responses.

When we first observed amiloride facilating plasmid DNA entry into cell lines (Fig. 1), we were interested in which uptake pathway amiloride might affect. Since amiloride has been used as a macropinocytosis inhibitor [16] and yet it facilitated DNA plasmid entering cells, other endocytic pathways were implied. Since inhibition of two other pathways of endocytosis by use of their respective inhibitors reduced the level of DNA plasmid entry, we deduce that lipid-raft- and caveolae-dependent endocytosis may be the main pathways involved (Fig. S1). However, amiloride has been found to affect the polymeric formation of cytoskeletons, involving actins or tubulins, and directly blocks the epithelial sodium channel [30], [31], [32], [33], [34]. Hence mechanisms other than the endocytosis might also be involved in the DNA entry facilitation.

Various strategies have been developed to transfect plasmid into cell lines in vitro [35], [36], including liposome-based transduction, but few has been successfully adapted in vivo [37], [38]. Higher transducing efficiency has been achieved by the use of in vivo electroporation and gen gun delivery [10], [11], [12], [13], however, those require special equipment and may cause some discomfort in vaccinees [14]. Therefore, since amiloride facilitated plasmid entry and expression in vitro, simple co-injection of amiloride with plasmid DNA was tested for its in vivo effects. Co-injection in vivo facilitated plasmid into APCs and expression of the encoded gene (Fig. 2). This demonstration of both in vitro and in vivo effects suggests benefits for both DNA vaccine technology in general as well as other applications such as gene and RNAi therapies [39]. There is also the possibility of combining amiloride and electroporation in vivo to improve DNA vaccine efficacy.

Naked DNA vaccine is routinely administrated by injection intramuscularly to take advantage of the large volume of muscle fibers to take in DNA and so to result in higher expression of the plasmid. However, muscle cells are not specialized as antigen presenting cells, and antigen expressed in muscle provides an inefficient stimulus to the immune responses compared to expression in APCs [40], [41], [42], [43], [44]. Lymph node injection technology has previously been demonstrated to enhance DNA vaccine induced immune response by 100-fold by favoring antigen expression in APCs [11] and the higher antigen expression correlated with higher levels of maturation of the transduced APCs [45]. The increased loading of epitopes from expressed antigen onto MHC presumably provided more efficient stimulation by the APCs to produce higher levels of pro-inflammatory cytokines [46]. Our findings are consistent with this view. Amiloride facilated plasmid entry and expression in APCs, thereby inducing higher levels of maturation and cytokine secretion in these treated cells (Fig. 3), whereas amiloride alone wasn't capable of promoting APCs' maturation (Fig. S2). These matured APCs could then affect adaptive immune responses so that higher levels of antibody production and cell-mediated responses were obtained as depicted in Figure 4. Antigen specific CTL killing can directly contribute to immunological function against, or clearing of chronic viral infections. Therefore it is significant that although the numbers of activated CD8 T cells were similar, (Fig. 5A), inclusion of amiloride induced significantly more CD8 CTL (Fig. 4D, 4E, 4F, 4G). The discrepancy was due to different functional CD8 T cells being induced. Thus the amiloride-induced CD8 CTL contained higher proportions of IFN-g^+^Perforin^+^GranzymeB^+^ (triple positive) cells (Fig. 5B, 5C, 5D). This differential induction has been further demonstrated by an in vitro study in which antigen+amiloride treated macrophages or splenocyte-DC had increased ability to convert antigen primed CD8 T cells into triple positive CD8+ T cells (Fig. 5E, F). These results indicated that amiloride is not only a facilitator for DNA entry and expression, but also a very important adjuvant to affect adaptive immune responses.

Thus, amiloride, a prescription drug for hypertension, could facilitate plasmid transduction of antigen presenting cells (APCs) both in vitro and in vivo. The transduced APCs could undergo enhanced maturation and cytokine production resulting in dramatically enhanced adaptive immune responses. Amiloride thus induced multi-functional CD8 CTL, which may lead to the development of novel therapeutic vaccination strategies against chronic infectious diseases. This facilitation by amiloride may be useful not only for DNA vaccination, but also for non-viral vector based gene therapy.

## Materials and Methods

### Animal, cell line and reagents

Adult female C57BL/6 mice (8–10 week of age) were from Beijing Vital Laboratory Animal Technology Company, Ltd. (Beijing, China) and kept in SPF condition. HBV sAg transgenic mice Alb1-HBV and IFN-γ^−/−^ mice (B6.129S7-Ifngtm1Ts/J) were purchased from Jackson Lab (Main, USA). All animal experiments were approved by the Committee of Experiment Animals of China Agricultural University. RAW264.7, JAWSII and DC2.4 were purchase from ATCC (VA, USA). Lipofactamine^TM^2000 was purchased from Invitrogen (CA, USA). HBsAg was obtained from NCPC Ltd. (Hebei, China). S208–215 peptide was synthesized by Scipeptide Ltd. (Shanghai, China). pcD-S2 was cloned and reserved in lab [47]. All antibodies for DC maturation (anti-CD40-PE, anti-CD80-PE, anti-CD83-PE, anti-CD86-PE, anti-MHC I-PE, anti-MHC II-PE), cell subset identification (anti-CD11c-FITC, anti-CD11c-APC, anti-CD11b-FITC, anti-CD11b-APC, anti-B220-PE, anti-CD3-FITC, anti-CD3-APC) and multi-color flow cytometry (anti-CD3-APC-Cy7, anti-CD8-FITC, anti-IFN-γ-PerCP-Cy5.5, anti-perforin-PE and anti-granzymeB-PE-Cy7) were purchased from eBioscience (CA, USA). Flexset kits for IL-6, TNF, IL-1β and IFN-γ were purchased from BD Biosciences (USA).

### Cell culture and inhibitor treatment

RAW264.7 and DC2.4 were cultured in DMEM/10%FCS, and JAWSII was cultured in DMEM/10%FCS with GM-CSF (1000 U/ml, Peprotech, USA). Amiloride (Sigma-Aldrich, USA) was prepared at 10 mM as stock solution and diluted to 1 mM, 100 uM and 10 uM in DMEM medium before treatments. After medium was removed, cells were treated with amiloride, MβCD (5 mM, Sigma) or Fillipin (10 µg/ml, Sigma) at 37°C for 1 h. The cells were treated with LPS (10 ng/ml, Sigma) or 10 µg/ml DNA in DMEM at 37°C for 0.5 h and washed 3 times. The culture medium was added back for continuing culture.

Peritoneal macrophage was washed from the murine peritoneal cavity with 10 ml PBS, rinsed and tested for F4/80 positivity. The F4/80+ macrophages were routinely present at a frequency of ∼50–70%. Spleen dendritic cells were prepared from plate-adhesive cells and purified with Miltenyi DC purification kit (Miltenyi Biotec, Gladbach, Germany). Cells were treated and cultured for 3 days for assessment of innate response.

### Plasmid preparation and fluorescence conjugation

pEGFP (Clontech, USA) and pcD-S2 plasmid were made as midi-preps from DH5α culture, purified by EndoFree Plasmid Maxi Kit (Qiagen, Germany) and endotoxin was below 10 EU/mg by LAL test. Cy5 was conjugated to plasmid with Mirus Label IT Kit (Mirus, USA) according to the manufacturer's instruction.

### Plasmid Entry Test

For *in vitro* testing, cell lines were treated with 1 mM amiloride and 10 µg/ml Cy5 conjugated pEGFP was added. Lipo2000 was added as positive control. Cy5+ cells were analyzed for plasmid entry at 2 hours later and GFP+ cells were analyzed for expression on day 2. For in vivo facilitation, 20 µg Cy5-pEGFP in PBS was injected into C57/B6 mice right hind footpad +/− amiloride. Both inguinal lymph nodes were collected for assessment of Cy5+ cell proportion 4 h later, or 24 h later for GFP expression. For induction of immune responses, 20 µg pcD-S2 in PBS was injected into hind footpad +/− amiloride 4 times at 2-week intervals.

### In vitro and in vivo CTL

*In vitro* CTL assay was performed as reported, with slight modifications [48]. Briefly, CD8+ T cells were purified from the splenocytes of immunized mice with a CD8 purification kit (R&D systems, USA) and used as effecter cells. Splenocytes from naïve C57BL/6 mice were pulsed with 10^−6^ M of HBsAg CTL peptide S208–215 [49] and labeled with 30 µM CFSE for use as target cells. The same naïve splenocytes without peptide pulse were labeled with 10 µM CFSE as control. Effecters and target cells were mixed at ratios of 10∶1, 1∶1 and 1∶10. After 3 days in culture, lysis of target cells was analyzed by FACSCalibur (BD Biosciences, USA). Specific lysis was calculated as (1−target cells/control cells)×100%.

*In vivo* CTL assay was performed as described previously [47], using S208–215 peptide and splenocytes from naïve C57BL/6 mice that had been labeled with 30 µM CFSE as target cells. Splenocytes without peptide were labeled 10 µM CFSE as control. The target and control cells were mixed in a 1∶1 ratio and i.v. injected into immunized mice at 2×10^7^ total cells per mouse. Splenocytes of injected mice were collected and analyzed 12 h later.

For Alb1-HBV mice, single cell suspensions were prepared from liver and the hepatic cells were used as target cells in in vitro and in vivo CTL assays as described above. For *in vivo* CTL assay, 10% CFSE cells were in spleen 12 h after transfer. Data was acquired until CFSE^low^ (10 µM) cells reached 10^4^.

### Multi-color Flow Cytometry

A multi-color antibody panel was set up with anti-CD3, anti-CD8, anti-IFN-γ, anti-perforin and anti-granzyme B. Splenocytes were re-stimulated *in vitro* by sAg for 24 h or S208–215 for 12 h, then after monensin block for 6 h, splenocyte were fixed and stained. Data was collected with BD Aria I and analyzed with Flowjo (Tree Star, Ashland, USA) and Grapher (Golden Software, USA).

pcD-S2 (10 µg/ml) with or without 100 µM amiloride was used to treat peritoneal macrophages or spleen dendritic cells in culture for 2 days. At day 3, purified CD8 T cells (R&D systems, USA) were added into the APC cultures at APC : T cell ratios of 1∶5, 1∶2 and 1∶1. At day 8, cells were washed with medium and re-stimulated overnight with S208–215 (10 µg/ml) or PMA+Ionomycin as positive controls. The re-stimulated cells were assayed after multi-color staining as mentioned above.

### Statistics

Data were analyzed using the one-tail Student's t-test (Fig. 3, 4E, 4G, 5D, E, F,), one-way ANOVA for more than 2 groups (Fig. 1, 2B, 4C, 5C, Fig. S1), or two-way ANOVA (Fig. 4D, 4F). Differences were considered to be statistically significant with p<0.05 for * and p<0.01 for **.

## Supporting Information

Figure S1 **Amiloride's acceleration of plasmid entry is lipid-raft and caveolae-dependent.** Lipid-raft inhibitor, MβCD, or caveolae inhibitor, fillipin was added with amiloride to block endocytosis pathways on cell lines, RAW264.7 (A, B), JAWSII (C, D), and DC2.4 (E, F). Then Cy5-pEGFP was added for assay of entry in 2 h and expression in 3 days. Shown is one of three independent experiments with similar results.
(TIF)

Figure S2 **Amiloride alone does not promote APC matuation.** Surface maturation markers CD40, CD80, CD83, CD86, MHC I and/or MHC II were tested at day 3 on RAW264.7 (A), JAWSII (B), DC2.4 (C), peritoneal macrophage (D) and spleno-DC (E), with or without 1 mM amiloride treatment. Shown is one of three independent experiments with similar results. For peritoneal macrophage and spleno-DC, n = 3. * and ** indicate significant difference between +/− amiloride.
(TIF)

Figure S3 **Amiloride enhances APC maturation.** Surface maturation markers CD40, CD80, CD83, CD86, MHC I and/or MHC II were tested at day 3 on RAW264.7 (A), JAWSII (B), DC2.4 (C), peritoneal macrophage (D) and spleno-DC (E), with or without 1 mM amiloride treatment. MFI data were showed. Shown is one of three independent experiments with similar results. For peritoneal macrophage and spleno-DC, n = 3. * and ** indicate significant difference between +/− amiloride.
(TIF)

Figure S4 **Amiloride increases multi-cytokine CD8 T cells.** Re-stimulated, monensin blocked CD8 T cells were stained and analyzed with gate hierarchy (A). Pie graph of cytokine expression from each mouse was showed, splenocytes re-stimulated with 10 µg/ml S208–215 for 12 h (B), or co-cultured with peritoneal macrophages followed by re-stimulation with S208–215 (C), or with spleno-DC (D) are also shown. Data represent three independent experiments with similar results.
(TIF)

## References

[1] Coney L, Wang B, Ugen KE, Boyer J, McCallus D (1994). Facilitated DNA inoculation induces anti-HIV-1 immunity in vivo.. Vaccine.

[2] Brown SE, Stanley C, Howard CR, Zuckerman AJ, Steward MW (1986). Antibody responses to recombinant and plasma derived hepatitis B vaccines.. Br Med J (Clin Res Ed).

[3] Donnelly JJ, Wahren B, Liu MA (2005). DNA vaccines: progress and challenges.. J Immunol.

[4] Donnelly JJ, Ulmer JB, Shiver JW, Liu MA (1997). DNA vaccines.. Annu Rev Immunol.

[5] Gurunathan S, Klinman DM, Seder RA (2000). DNA vaccines: immunology, application, and optimization*.. Annu Rev Immunol.

[6] Fynan EF, Webster RG, Fuller DH, Haynes JR, Santoro JC (1993). DNA vaccines: protective immunizations by parenteral, mucosal, and gene-gun inoculations.. Proc Natl Acad Sci U S A.

[7] Rocha-Zavaleta L, Alejandre JE, Garcia-Carranca A (2002). Parenteral and oral immunization with a plasmid DNA expressing the human papillomavirus 16-L1 gene induces systemic and mucosal antibodies and cytotoxic T lymphocyte responses.. J Med Virol.

[8] Belperron AA, Feltquate D, Fox BA, Horii T, Bzik DJ (1999). Immune responses induced by gene gun or intramuscular injection of DNA vaccines that express immunogenic regions of the serine repeat antigen from Plasmodium falciparum.. Infect Immun.

[9] Leitner WW, Baker MC, Berenberg TL, Lu MC, Yannie PJ (2009). Enhancement of DNA tumor vaccine efficacy by gene gun-mediated codelivery of threshold amounts of plasmid-encoded helper antigen.. Blood.

[10] Hirao LA, Wu L, Khan AS, Satishchandran A, Draghia-Akli R (2008). Intradermal/subcutaneous immunization by electroporation improves plasmid vaccine delivery and potency in pigs and rhesus macaques.. Vaccine.

[11] Smith KA, Tam VL, Wong RM, Pagarigan RR, Meisenburg BL (2009). Enhancing DNA vaccination by sequential injection of lymph nodes with plasmid vectors and peptides.. Vaccine.

[12] Widera G, Austin M, Rabussay D, Goldbeck C, Barnett SW (2000). Increased DNA vaccine delivery and immunogenicity by electroporation in vivo.. J Immunol.

[13] Aberle JH, Aberle SW, Allison SL, Stiasny K, Ecker M (1999). A DNA immunization model study with constructs expressing the tick-borne encephalitis virus envelope protein E in different physical forms.. J Immunol.

[14] Pilling AM, Harman RM, Jones SA, McCormack NA, Lavender D (2002). The assessment of local tolerance, acute toxicity, and DNA biodistribution following particle-mediated delivery of a DNA vaccine to minipigs.. Toxicol Pathol.

[15] Wang B, Ugen KE, Srikantan V, Agadjanyan MG, Dang K (1993). Gene inoculation generates immune responses against human immunodeficiency virus type 1.. Proc Natl Acad Sci U S A.

[16] Koivusalo M, Welch C, Hayashi H, Scott CC, Kim M Amiloride inhibits macropinocytosis by lowering submembranous pH and preventing Rac1 and Cdc42 signaling.. J Cell Biol.

[17] Gong Q, Weide M, Huntsman C, Xu Z, Jan LY (2007). Identification and characterization of a new class of trafficking motifs for controlling clathrin-independent internalization and recycling.. J Biol Chem.

[18] Hood SJ, Taylor KP, Ashby MJ, Brown MJ (2007). The spironolactone, amiloride, losartan, and thiazide (SALT) double-blind crossover trial in patients with low-renin hypertension and elevated aldosterone-renin ratio.. Circulation.

[19] Rejman J, Conese M, Hoekstra D (2006). Gene transfer by means of lipo- and polyplexes: role of clathrin and caveolae-mediated endocytosis.. J Liposome Res.

[20] Ignatovich IA, Dizhe EB, Pavlotskaya AV, Akifiev BN, Burov SV (2003). Complexes of plasmid DNA with basic domain 47–57 of the HIV-1 Tat protein are transferred to mammalian cells by endocytosis-mediated pathways.. J Biol Chem.

[21] Qian ZM, Li H, Sun H, Ho K (2002). Targeted drug delivery via the transferrin receptor-mediated endocytosis pathway.. Pharmacol Rev.

[22] Di Fabio S, Mbawuike IN, Kiyono H, Fujihashi K, Couch RB (1994). Quantitation of human influenza virus-specific cytotoxic T lymphocytes: correlation of cytotoxicity and increased numbers of IFN-gamma producing CD8+ T cells.. Int Immunol.

[23] Wan Y, Lu L, Bramson JL, Baral S, Zhu Q (2001). Dendritic cell-derived IL-12 is not required for the generation of cytotoxic, IFN-gamma-secreting, CD8(+) CTL in vivo.. J Immunol.

[24] Chattopadhyay PK, Betts MR, Price DA, Gostick E, Horton H (2009). The cytolytic enzymes granyzme A, granzyme B, and perforin: expression patterns, cell distribution, and their relationship to cell maturity and bright CD57 expression.. J Leukoc Biol.

[25] Johnson BJ, Costelloe EO, Fitzpatrick DR, Haanen JB, Schumacher TN (2003). Single-cell perforin and granzyme expression reveals the anatomical localization of effector CD8+ T cells in influenza virus-infected mice.. Proc Natl Acad Sci U S A.

[26] Shacklett BL, Cox CA, Quigley MF, Kreis C, Stollman NH (2004). Abundant expression of granzyme A, but not perforin, in granules of CD8+ T cells in GALT: implications for immune control of HIV-1 infection.. J Immunol.

[27] Makedonas G, Hutnick N, Haney D, Amick AC, Gardner J (2010). Perforin and IL-2 upregulation define qualitative differences among highly functional virus-specific human CD8 T cells.. PLoS Pathog.

[28] Makedonas G, Banerjee PP, Pandey R, Hersperger AR, Sanborn KB (2009). Rapid up-regulation and granule-independent transport of perforin to the immunological synapse define a novel mechanism of antigen-specific CD8+ T cell cytotoxic activity.. J Immunol.

[29] Blackburn SD, Shin H, Haining WN, Zou T, Workman CJ (2009). Coregulation of CD8+ T cell exhaustion by multiple inhibitory receptors during chronic viral infection.. Nat Immunol.

[30] Jeng RL, Welch MD (2001). Cytoskeleton: actin and endocytosis–no longer the weakest link.. Curr Biol.

[31] Qualmann B, Kessels MM (2002). Endocytosis and the cytoskeleton.. Int Rev Cytol.

[32] Lanzetti L, Di Fiore PP, Scita G (2001). Pathways linking endocytosis and actin cytoskeleton in mammalian cells.. Exp Cell Res.

[33] Ondrej V, Lukasova E, Falk M, Kozubek S (2007). The role of actin and microtubule networks in plasmid DNA intracellular trafficking.. Acta Biochim Pol.

[34] Geiger RC, Taylor W, Glucksberg MR, Dean DA (2006). Cyclic stretch-induced reorganization of the cytoskeleton and its role in enhanced gene transfer.. Gene Ther.

[35] Yang Z, Sahay G, Sriadibhatla S, Kabanov AV (2008). Amphiphilic block copolymers enhance cellular uptake and nuclear entry of polyplex-delivered DNA.. Bioconjug Chem.

[36] Vandenbroucke RE, Lucas B, Demeester J, De Smedt SC, Sanders NN (2007). Nuclear accumulation of plasmid DNA can be enhanced by non-selective gating of the nuclear pore.. Nucleic Acids Res.

[37] Karkada M, Weir GM, Quinton T, Fuentes-Ortega A, Mansour M (2010). A liposome-based platform, VacciMax, and its modified water-free platform DepoVax enhance efficacy of in vivo nucleic acid delivery.. Vaccine.

[38] Nomura T, Nakajima S, Kawabata K, Yamashita F, Takakura Y (1997). Intratumoral pharmacokinetics and in vivo gene expression of naked plasmid DNA and its cationic liposome complexes after direct gene transfer.. Cancer Res.

[39] Lohr F, Lo DY, Zaharoff DA, Hu K, Zhang X (2001). Effective tumor therapy with plasmid-encoded cytokines combined with in vivo electroporation.. Cancer Res.

[40] Haddad D, Ramprakash J, Sedegah M, Charoenvit Y, Baumgartner R (2000). Plasmid vaccine expressing granulocyte-macrophage colony-stimulating factor attracts infiltrates including immature dendritic cells into injected muscles.. J Immunol.

[41] Donnelly JJ, Liu MA, Ulmer JB (2000). Antigen presentation and DNA vaccines.. Am J Respir Crit Care Med.

[42] Paul L, Porgador A (2000). DNA-based vaccines: role of dendritic cells in antigen presentation.. Adv Exp Med Biol.

[43] Rice J, King CA, Spellerberg MB, Fairweather N, Stevenson FK (1999). Manipulation of pathogen-derived genes to influence antigen presentation via DNA vaccines.. Vaccine.

[44] Feltquate DM (1998). DNA vaccines: vector design, delivery, and antigen presentation.. J Cell.

[45] Weissman D, Ni H, Scales D, Dude A, Capodici J (2000). HIV gag mRNA transfection of dendritic cells (DC) delivers encoded antigen to MHC class I and II molecules, causes DC maturation, and induces a potent human in vitro primary immune response.. J Immunol.

[46] Shedlock DJ, Weiner DB (2000). DNA vaccination: antigen presentation and the induction of immunity.. J Leukoc Biol.

[47] Du X, Zheng G, Jin H, Kang Y, Wang J (2007). The adjuvant effects of co-stimulatory molecules on cellular and memory responses to HBsAg DNA vaccination.. J Gene Med.

[48] Piriou L, Chilmonczyk S, Genetet N, Albina E (2000). Design of a flow cytometric assay for the determination of natural killer and cytotoxic T-lymphocyte activity in human and in different animal species.. Cytometry.

[49] Schirmbeck R, Stober D, El-Kholy S, Riedl P, Reimann J (2002). The immunodominant, Ld-restricted T cell response to hepatitis B surface antigen (HBsAg) efficiently suppresses T cell priming to multiple Dd-, Kd-, and Kb-restricted HBsAg epitopes.. J Immunol.

