# Supplementary figures and images for: Amiloride Enhances Antigen Specific CTL by Faciliting HBV DNA Vaccine Entry into Cells
Source: PLoS One. 2012 Mar 16;7(3):e33015. doi: 10.1371/journal.pone.0033015 (PMC3306379; doi:10.1371/journal.pone.0033015)

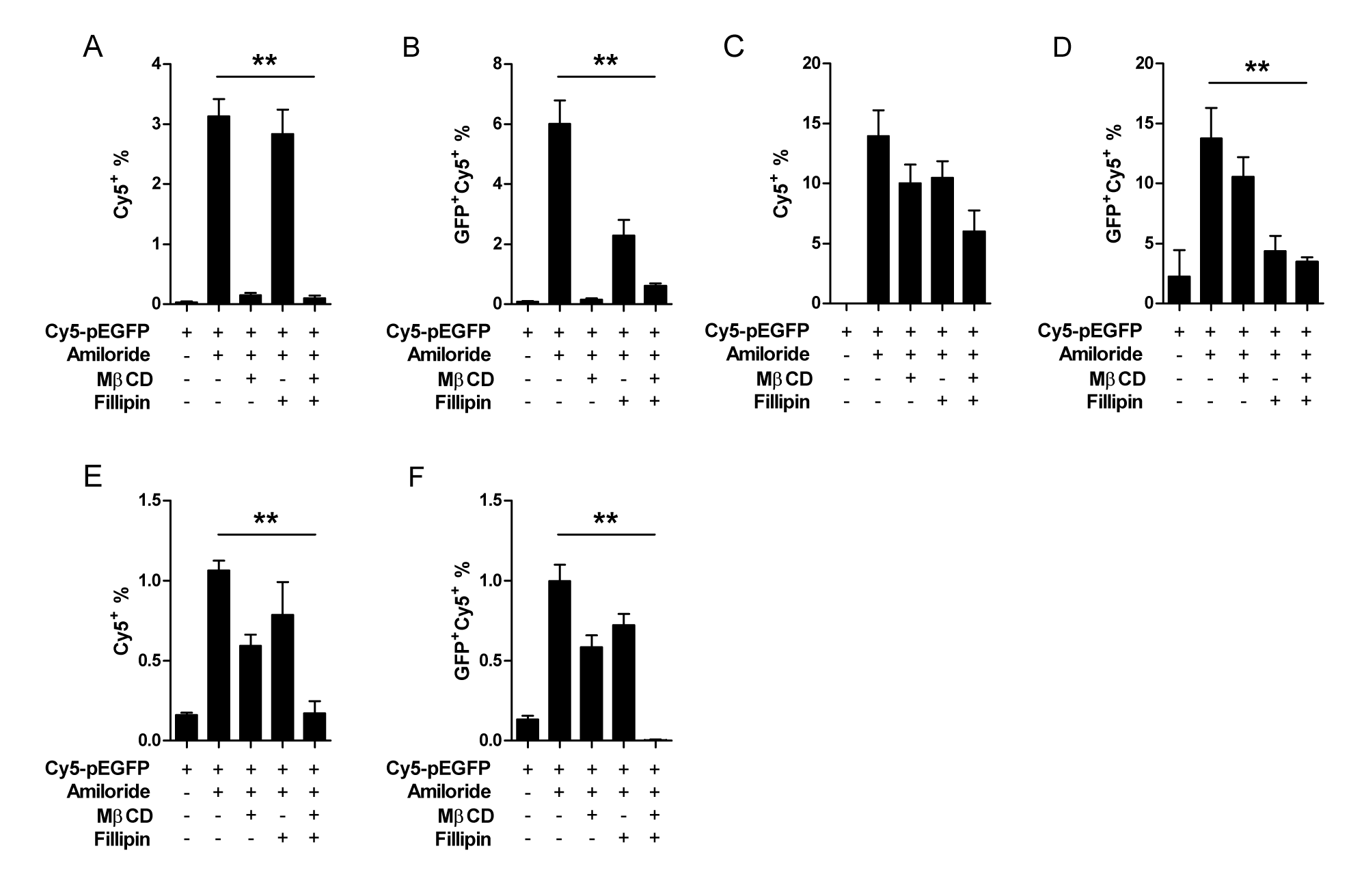

Supplement: Figure S1 — Amiloride's acceleration of plasmid entry is lipid-raft and caveolae-dependent. Lipid-raft inhibitor, MβCD, or caveolae inhibitor, fillipin was added with amiloride to block endocytosis pathways on cell lines, RAW264.7 (A, B), JAWSII (C, D), and DC2.4 (E, F). Then Cy5-pEGFP was added for assay of entry in 2 h and expression in 3 days. Shown is one of three independent experiments with similar results. (TIF) [file pone.0033015.s001.tif]

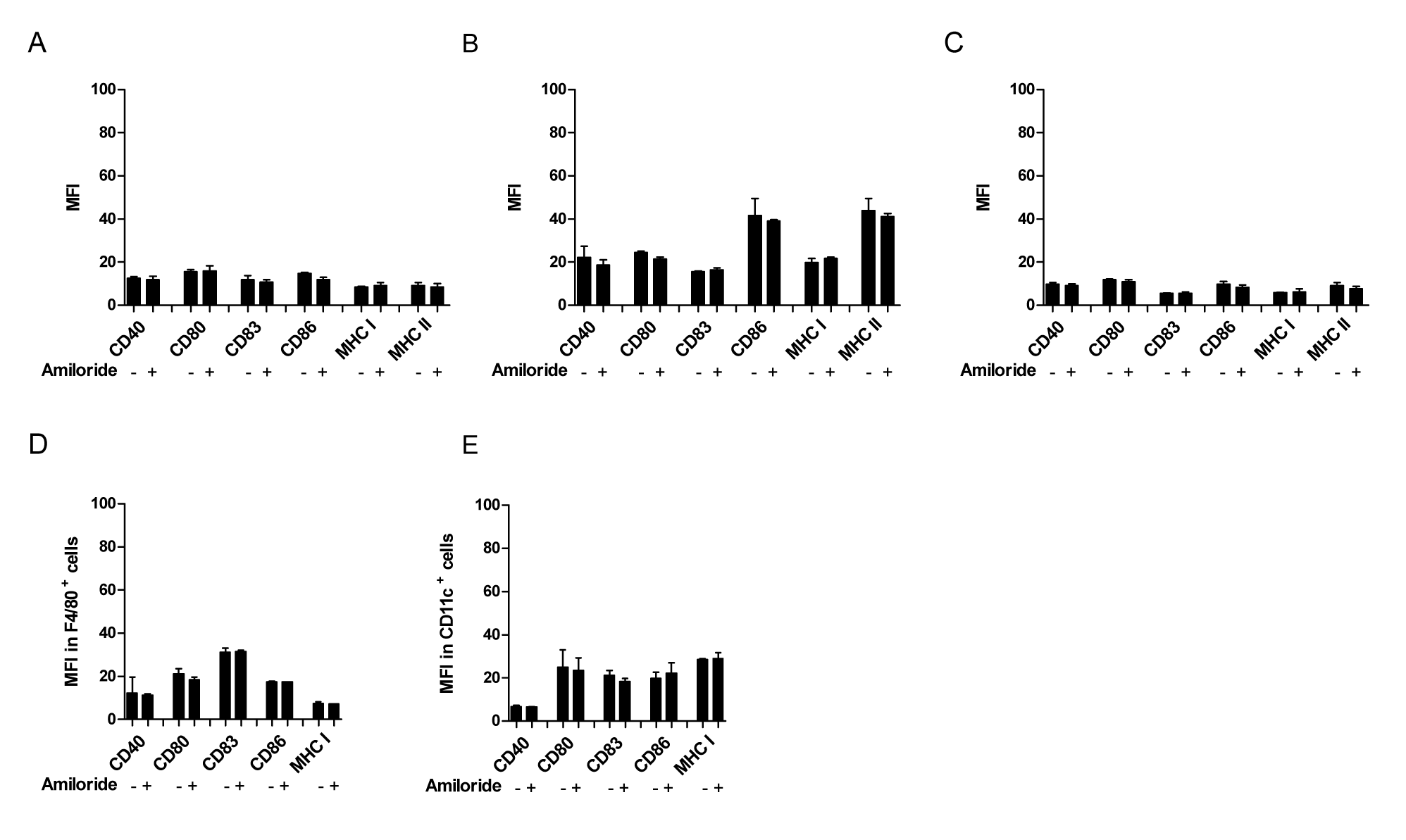

Supplement: Figure S2 — Amiloride alone does not promote APC matuation. Surface maturation markers CD40, CD80, CD83, CD86, MHC I and/or MHC II were tested at day 3 on RAW264.7 (A), JAWSII (B), DC2.4 (C), peritoneal macrophage (D) and spleno-DC (E), with or without 1 mM amiloride treatment. Shown is one of three independent experiments with similar results. For peritoneal macrophage and spleno-DC, n = 3. * and ** indicate significant difference between +/− amiloride. (TIF) [file pone.0033015.s002.tif]

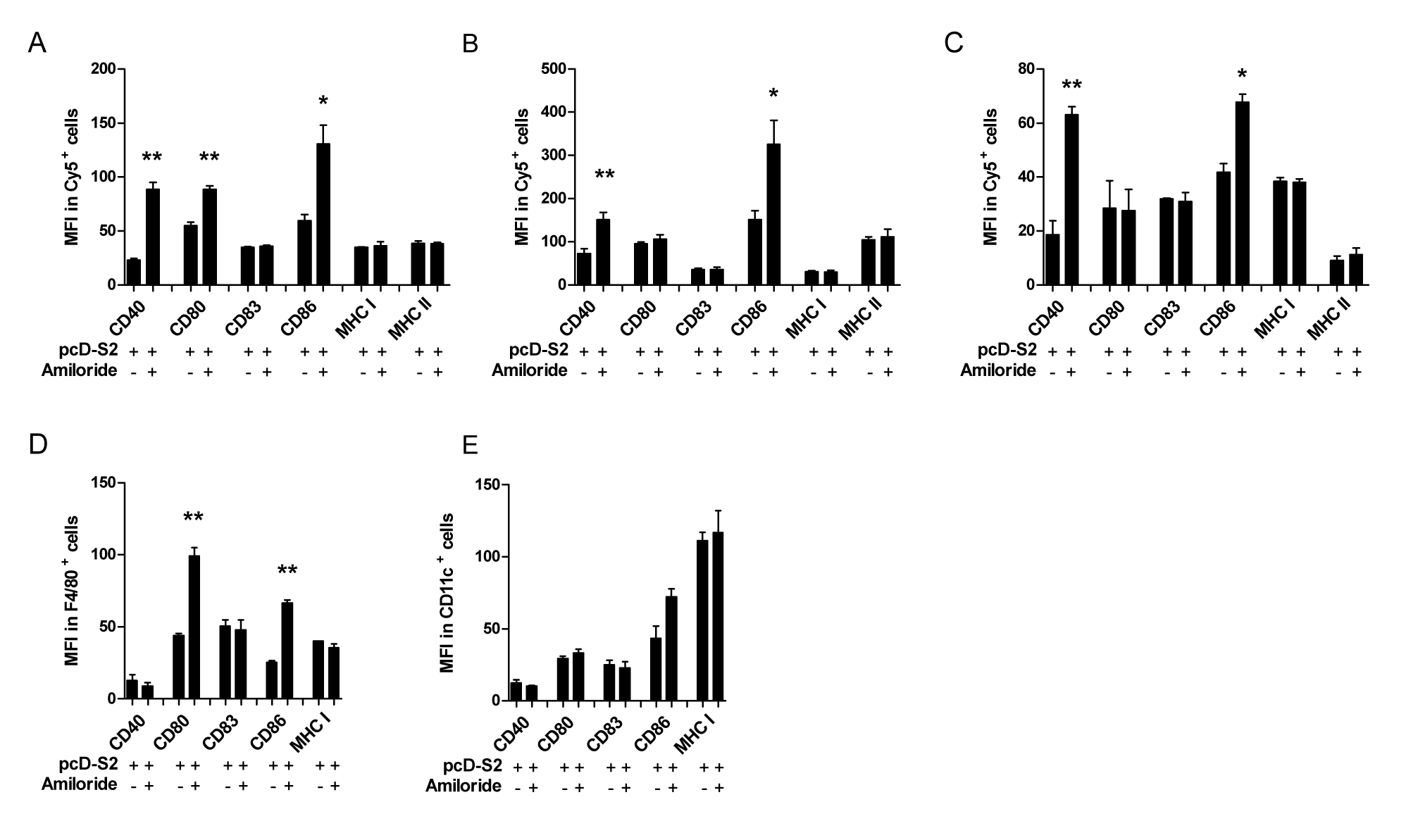

Supplement: Figure S3 — Amiloride enhances APC maturation. Surface maturation markers CD40, CD80, CD83, CD86, MHC I and/or MHC II were tested at day 3 on RAW264.7 (A), JAWSII (B), DC2.4 (C), peritoneal macrophage (D) and spleno-DC (E), with or without 1 mM amiloride treatment. MFI data were showed. Shown is one of three independent experiments with similar results. For peritoneal macrophage and spleno-DC, n = 3. * and ** indicate significant difference between +/− amiloride. (TIF) [file pone.0033015.s003.tif]

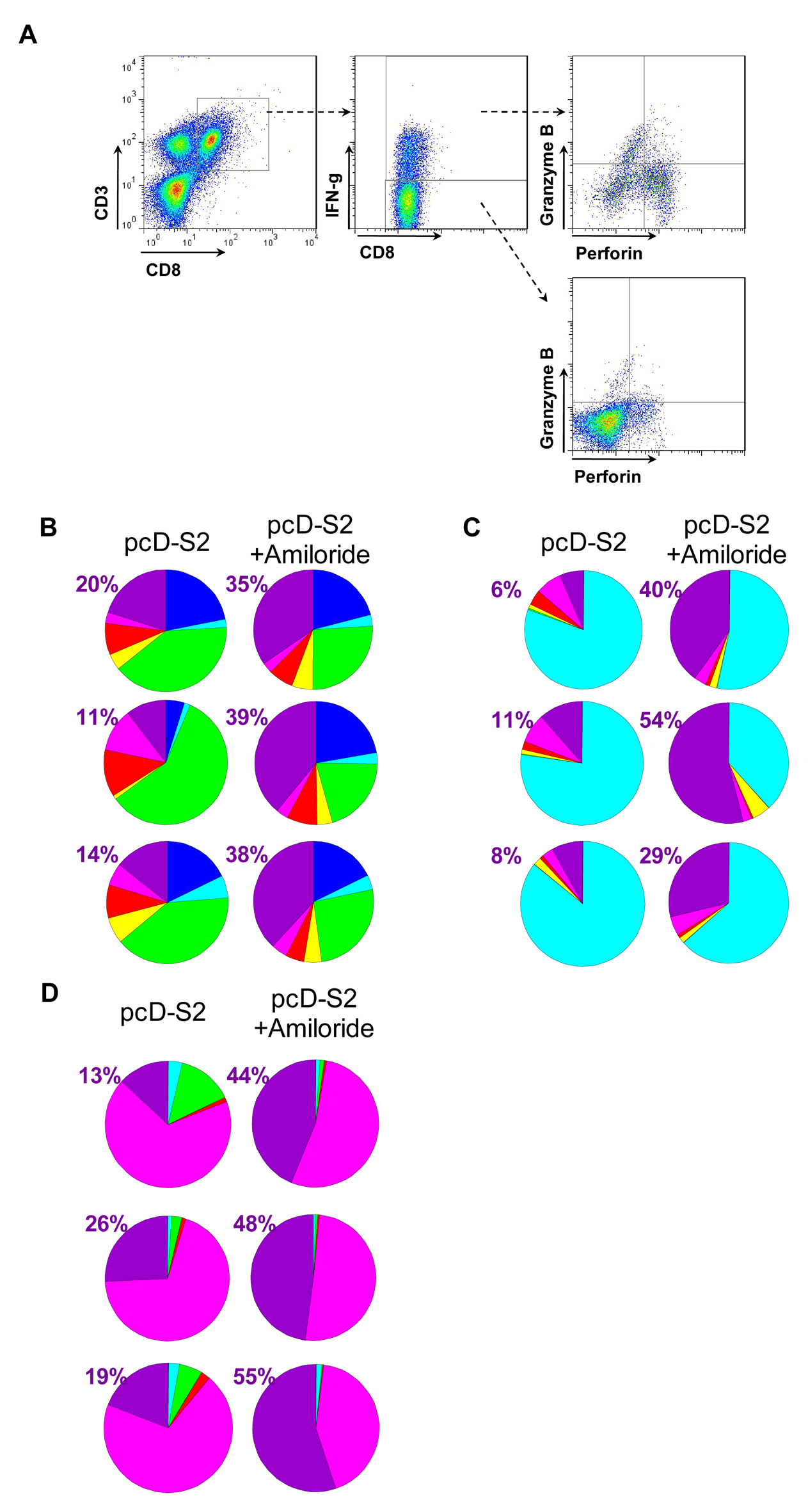

Supplement: Figure S4 — Amiloride increases multi-cytokine CD8 T cells. Re-stimulated, monensin blocked CD8 T cells were stained and analyzed with gate hierarchy (A). Pie graph of cytokine expression from each mouse was showed, splenocytes re-stimulated with 10 µg/ml S208–215 for 12 h (B), or co-cultured with peritoneal macrophages followed by re-stimulation with S208–215 (C), or with spleno-DC (D) are also shown. Data represent three independent experiments with similar results. (TIF) [file pone.0033015.s004.tif]
